# Diagnostic evaluation of three cardiac software packages using a consecutive group of patients

**DOI:** 10.1186/2191-219X-1-22

**Published:** 2011-09-30

**Authors:** Lena Johansson, Milan Lomsky, Jens Marving, Mattias Ohlsson, Sven-Eric Svensson, Lars Edenbrandt

**Affiliations:** 1 Department of Molecular and Clinical Medicine, Clinical Physiology, Sahlgrenska University Hospital, Gothenburg, Sweden; 2 Department of Clinical Sciences, Malmö, Lund University, Lund, Sweden; 3 Department of Theoretical Physics, Lund University, Lund, Sweden; 4 Department of Clinical Physiology, Skåne University Hospital, Malmö, 205 02 Malmö, Sweden

**Keywords:** myocardial perfusion imaging, SPECT, automatic quantification, software, coronary artery disease

## Abstract

**Purpose:**

The aim of this study was to compare the diagnostic performance of the three software packages 4DMSPECT (4DM), Emory Cardiac Toolbox (ECTb), and Cedars Quantitative Perfusion SPECT (QPS) for quantification of myocardial perfusion scintigram (MPS) using a large group of consecutive patients.

**Methods:**

We studied 1,052 consecutive patients who underwent 2-day stress/rest 99mTc-sestamibi MPS studies. The reference/gold-standard classifications for the MPS studies were obtained from three physicians, with more than 25 years each of experience in nuclear cardiology, who re-evaluated all MPS images. Automatic processing was carried out using 4DM, ECTb, and QPS software packages. Total stress defect extent (TDE) and summed stress score (SSS) based on a 17-segment model were obtained from the software packages. Receiver-operating characteristic (ROC) analysis was performed.

**Results:**

A total of 734 patients were classified as normal and the remaining 318 were classified as having infarction and/or ischemia. The performance of the software packages calculated as the area under the SSS ROC curve were 0.87 for 4DM, 0.80 for QPS, and 0.76 for ECTb (QPS vs. ECTb *p *= 0.03; other differences *p *< 0.0001). The area under the TDE ROC curve were 0.87 for 4DM, 0.82 for QPS, and 0.76 for ECTb (QPS vs. ECTb *p *= 0.0005; other differences *p *< 0.0001).

**Conclusion:**

There are considerable differences in performance between the three software packages with 4DM showing the best performance and ECTb the worst. These differences in performance should be taken in consideration when software packages are used in clinical routine or in clinical studies.

## Introduction

Visual interpretation of myocardial perfusion scintigrams (MPS) is dependent on the experience and knowledge of the physician, and subject to inter- and intraobserver variability [1]. Software packages for automated quantification of MPS have been developed in order to make the interpretations more standardized. Modules of these packages for automatic assessment of left ventricular function from a gated MPS have been extensively compared [2-4]. The corresponding modules for automatic quantification of the perfusion of the left ventricle have only been compared in a limited number of studies [5-7].

The most widely used approach to quantifying perfusion is to divide the left ventricular myocardium into 17 or 20 segments and to score each segment for perfusion defects using a five-point scale [8]. The sum of the scores in the segments for the stress images are defined as the summed stress score (SSS). This standard parameter is provided in a consistent manner by the three software packages 4D-MSPECT (4DM, Invia Medical Imaging Solutions, Ann Arbor, MI, USA) [9], Emory Cardiac Toolbox (ECTb, Emory University, Atlanta, GA, USA) [10] and Quantitative Perfusion SPECT (QPS, Cedars-Sinai Medical Center, Los Angeles, CA, USA) [11] to mimic visual reading. Considerable variability between SSS values obtained with the different software packages has been reported [5-7].

The total stress defect extent (TDE) of 3% or greater has also been proposed as a criterion for abnormality by the group behind ECTb [10]. The extent of the left ventricle being hypoperfused is provided by all three software packages and also possible to compare.

Guner et al. found substantial differences in magnitudes of the SSS and TDE values produced by 4DM, ECTb, and QPS, indicating that different thresholds need to be applied to the different software packages [7]. Receiver-operating characteristic (ROC) analysis as well as comparisons of specificities of the software packages at similar levels of sensitivity therefore needs to be performed in order to compare the diagnostic performances.

The purpose of this study was to compare the diagnostic performance of the three software packages 4DM, ECTb, and QPS for quantification of MPS using a large group of consecutive patients. All MPS studies were classified by three physicians with long experience of nuclear cardiology.

## Materials and methods

### Patients

The patients were selected from 1,245 consecutive patients who underwent rest/stress (exercise/adenosine)-gated MPS from September 15, 2005 to September 14, 2007 at the Sahlgrenska University Hospital, Gothenburg, Sweden. Patients with incomplete data (missing rest, stress, or gated study) were not considered and only one examination per patient was included. A total of 100 patients with left-bundle branch block, or paced rhythm were excluded. Thirtu-four MPS studies of an insufficient technical quality, e.g., arrhythmia and inadequate level of exercise, and 59 studies of an insufficient image quality, e.g., high extra-cardiac uptake, were excluded. The study group comprised the remaining 1,052 patients. The clinical characteristics of these patients are summarized in Table 1. The study was approved by the Research Ethics Committee at Gothenburg University.

**Table 1:** Patient characteristics (*n *= 1,052)

Characteristic	Number
Age (years) mean ± SD	62 ± 10.3 (range 29 to 89)
Gender	
Female	553 (53%)
Male	499 (47%)
Body mass index (kg/m^2^) mean ± SD	26.6 ± 4.4
Body mass index > 30	185 (18%)
Chest pain	293 (28%)
Hypertension	551 (52%)
Diabetes	185 (18%)
Hypercholesterolemia	475 (45%)
Smoker	144 (14%)
Family history	375 (36%)
Infarction	147 (14%)
PTCA	149 (14%)
CABG	101 (10%)
Stress	
Adenosine	599 (57%)
Exercise	453 (43%)
Indication	
Diagnosis	788 (75%)
Known CAD	252 (24%)
Other	12 (1%)

### Stress testing

Patients were stressed using either pharmacological stress with adenosine (57%) or maximal symptom limited exercise on a bicycle ergometer (43%). The pharmacological stress or exercise was continued for at least 2 min after injection of the tracer.

### Imaging protocols

The gated single-photon emission computed tomography (SPECT) studies were performed using a 2-day non-gated stress/gated rest ^99 m^Tc-sestamibi protocol. Stress and rest acquisition began about 60 min after the injection of 600 MBq ^99 m^Tc-sestamibi. Images were acquired using two different dual-head SPECT cameras (Infinia or Millenium VG, General Electric, Fairfield, CT, USA) equipped with low-energy, high-resolution collimators. Acquisition was carried out in the supine position in step and shoot mode using circular acquisition and a 64 × 64 matrix, a zoom factor of 1.28 and a pixel size of 6.9 mm, with 60 projections over 180° and 40 s per projection. In patients weighing over 90 kg, the acquisition time per projection was increased to 55 s. During the rest acquisition, the patient was monitored using a three-lead ECG. The acceptance window was opened to ± 20% of the predefined R-R interval. Other beats were rejected. Each R-R interval was divided into eight equal time intervals. Gated-SPECT acquisition was performed at the same time as ungated routine SPECT acquisition. An automatic motion-correction program was applied in studies showing patient motion during acquisition.

### Tomographic reconstruction

Tomographic reconstruction was performed using filtered back-projection with a Butterworth filter for all studies. During the study period, the critical frequency and order were changed in from 0.40 to 0.52 cycles/cm and from order 10 to 5. The reconstruction of gated data used filtered back projection with a Butterworth filter with a critical frequency of 0.40 cycles/cm and order 10 for all studies. No attenuation or scatter correction was used.

### Reference classifications

The reference classifications for the MPS studies regarding presence or absence of ischemia and/or infarction were obtained from three physicians, each of whom had over 25 years' experience of nuclear cardiology. They re-evaluated all MPS images separately. All cases were classified visually, and a custom display software was developed for this purpose, allowing the experts to view slice images (short axis, horizontal and vertical long axis) of the rest, stress, and gated-rest studies, polar plots (rest, stress, rest-stress difference, stress/rest ratio, motion and thickening) and 3D-images. Color scales and contrast levels were adjustable. No quantitative results from any software package were available during the re-evaluation. The custom display software used images directly from the reconstructed data, i.e., polar maps and 3D images were not taken from any of the quantitation software packages in order to avoid that a clinician's familiarity with interpreting data from a particular package could influence the results.

The following clinical information was available during the re-evaluation process: age, gender, previous myocardial infarction, previous re-vascularisation, present smoking, presence of hypertension, hyperlipidemia, diabetes, peripheral vascular disease, positive family history, and presence of typical chest pain. At the time of the MPS, all patients were asked about clinical risk factors and the presence of symptoms. Clinical information was also collected from the referral cards.

The three experts classified each patient case separately, and the majority rule was applied in cases of disagreement, i.e. the reference classification of ischemia required that at least two of the three experts classified that case as ischemia. The experts also had the possibility of classifying an MPS as of insufficient quality, and these cases were excluded.

### Software packages

The following three software packages were used:

• 4D-MSPECT, Version 4.0, University of Michigan Medical Center (4DM) [9];

• Emory Cardiac Toolbox, Version 3.0, Emory University Medical Center (ECTb) [10];

• Cedars Quantitative Perfusion SPECT, Version 4.0, Cedars-Sinai Medical Center (QPS) [11].

Using the database menu in each package, databases that matched the described acquisition protocol were selected. Quantitative analysis was performed on a Xeleris workstation Version 2.0551 (General Electric, USA) for the 4DM, ECTb, and QPS packages. The same reconstructed short-axis images were loaded to all three software packages. Experienced laboratory technologists, blind to the reference classifications, processed the studies and manually corrected the automatic left ventricular positioning within each software package when necessary. Corrections were made only for major discrepancies, to avoid any unnecessary manipulation of the data. This approach was used in order to make the results relevant to other MPS clinics and not influenced by the opinion of our technologies.

The TDE and SSS values based on a 17-segment model were obtained from the software packages. ROC analysis was performed for the analysis of performance regarding the classification of the MPS studies as normal vs. abnormal (infarction and/or ischemia).

A commonly used criterion for abnormality is an SSS of 4 or greater. This criterion was originally used for 20-segment analysis. In this study, we used the currently recommended 17-segment model, which may produce slightly lower SSS values. We therefore also included the criterion SSS 3 or greater for abnormality. Furthermore, ECTb has shown to produce higher SSS values and we therefore also added a criterion SSS of 5 or greater. ECTb proposes a criterion for abnormality for TDE of 3% or greater [10]. All this criteria were evaluated.

### Statistical analysis

The significance of the difference between two obtained ROC areas was calculated using a permutation test [12]. The test is performed by repeatedly and randomly permuting the cases in the two lists. For each permutation the difference of the two resulting areas were calculated (test statistic). The evidence against the null hypothesis, of no difference between the two original ROC areas, was given by the fraction of area differences of the test statistic larger than the actual difference.

The significance of a difference in specificity or sensitivity between two software packages was tested, paying particular attention to the fact that the same studies were used, i.e., a McNemar type of statistic was used.

## Results

The contours required adjustment in 21 (2.0%), 32 (3.0%), and 9 (0.9%) of the 1,052 patients using the 4DM, ECTb, and QPS software packages, respectively.

The three experts' classifications showed ischemia in 257 patients and infarction in 150 patients. Eighty-nine of these patients had both ischemia and infarction, and the number of patients with either ischemia or infarction or both was 318. The remaining 734 patients were classified as normal. All three experts agreed regarding ischemia/no ischemia in 748 (71%) and regarding infarction/no infarction in 872 (83%) of the 1,052 patients.

The performances for the three software packages calculated as the areas under the ROC curves are presented in Table 2. For both SSS and TDE, 4DM showed better performance than QPS which showed better performance than ECTb, with all differences being statistically significant. For QPS, the TDE performance was slightly better than for the SSS, but for 4DM and ECTb there were no significant differences between TDE and SSS.

**Table 2:** The areas under the ROC curves for detection of perfusion abnormality

	4DM	ECTb	QPS	*p*
SSS	0.87 (0.85 - 0.89)	0.76 (0.73 *- *0.79)	0.80 (0.77 *- *0.82)	ECTb vs QPS *p *= 0.03
				All others *p *< 0.001
TDE	0.87 (0.85 *- *0.89)	0.76 (0.73 *- *0.79)	0.82 (0.79 *- *0.84)	All *p *< 0.001

Difference between SSS and TDE was significant (*p *= 0.03) for QPS and not significant for 4DM and ECTb.

The SSS criterion for abnormality of 3 or greater for 4DM and QPS showed approximately the same sensitivity as an SSS criterion of 5 or greater for ECTb (78.3% for 4DM; 76.1% for ECTb; 75.2% for QPS; differences not significant). The corresponding specificities were 80.2% for 4DM, 72.9 for QPS and 61.3% for ECTb (*p *= 0.0005 for QPS vs 4DM and *p *< 0.0001 for the other differences).

The TDE criterion of 3% or greater showed sensitivities of 87.4% for ECTb, 80.8% for 4DM, and 78.9% for QPS (*p *= 0.004 for ECTb vs 4DM; *p *= 0.0003 for ECTb vs QPS; not significant for 4DM vs QPS). The corresponding specificities were 79.3% for 4DM, 66.6% for QPS, and 41.6% for ECTb (all differences *p *< 0.0001) (Table 3).

**Table 3:** Comparison of the specificities and sensitivities for different SSS and TDE criteria

	4DM	ECTb	QPS
Specificity (*n *= 734)			
SSS < 3	80.2%	44.4%	72.9%
SSS < 4	86.1%	52.6%	79.8%
SSS < 5	89.5%	61.3%	85.3%
TDE < 3	79.3%	41.6%	66.6%
Sensitivity (*n *= 318)			
SSS ≥ 3	78.3%	85.5%	75.2%
SSS ≥ 4	72.3%	81.8%	66.7%
SSS ≥ 5	68.2%	76.1%	59.7%
TDE ≥ 3	80.8%	87.4%	78.9%

**Figure 1:**
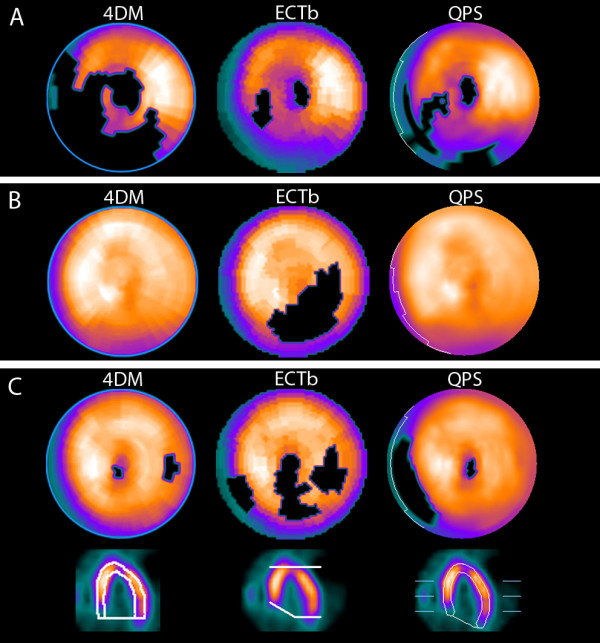
**Case illustration of three patients**. The polar map for the 4DM software is shown at left, for the ECTb software at center, and for the QPS software at right. (**A) **Abnormal case from a 58-year-old hypertensive man with typical chest pain. The summed stress score (SSS)/total stress defect extent (TDE) = 21/47%, 4/7%, and 8/14% for 4DM, ECTb, and QPS, respectively. (**B) **Normal case from a 68-year-old hypertensive woman with atypical chest pain. The SSS/TDE = 0/0%, 11/21%, and 0/0% for 4DM, ECTb, and QPS, respectively. (**C**) 74-year-old hypertensive woman with atypical chest pain and no risk factors. The SSS/TDE = 3/2%, 9/22%, and 8/8% for 4DM, ECTb, and QPS, respectively. The boundaries of the left ventricle automatically defined by the software packages are illustrated in the horizontal long axis slices. Note the different approach to define the septal part.

Figure 1 provides polar maps of three examples that reflect our main findings. The abnormal case (A) is true positive for all three packages but with differences in SSS between 4 and 21. The first normal case (B) is true negative for 4DM and QPS but clearly false positive for ECTb (SSS = 11). The second normal case (C) is false positive for both ECTb and QPS.

## Discussion

The results of this study show significant differences in diagnostic performance between the software packages for quantitative MPS analysis. Differences between the scores presented by 4DM, ECTb, and QPS have also been shown in previous studies. In the study of Wolak et al., 4DM and QPS also showed significantly higher performance than ECTb in the detection of coronary artery disease measured as area under the SSS ROC curve [6]. ECTb showed an area under the SSS ROC curve of 0.76 both in this study and that of Wolak et al. [6] and the sensitivity/specificity for the SSS criterion of 4 or greater for abnormality was similar in the two studies (82%/53% in this study; 85%/49% [6]). The relation between 4DM and QPS was different in the two studies. 4DM showed significantly higher area under the ROC curve than QPS while there was no significant difference in the study of Wolak et al. [6].

Guner et al. also studied the performance of 4DM, ECTb, and QPS in detecting coronary artery disease using MPS with ^201^Tl [7]. They did not find significant differences between the three software packages measured as area under the SSS or TDE ROC curves [7].

### Patient population

To the best of our knowledge, this is the largest study to compare MPS software packages based on material from consecutive MPS patients. We only excluded patients with paced rhythm or left-bundle branch block, or with technically insufficient studies, leaving 84% of the patients in the study population. The exclusion criteria are also less likely to bias the material towards more normal or abnormal cases. In contrast, Wolak et al. [6] only included 13% of the study group (328 out of 2,450) and Guner et al. [7] included 12% (283 out of 2,430) of the patients referred for MPS. In both these studies, they included MPS patients who had a coronary angiogram within 60 days or 3 months of the MPS study. Wolak [6] also included a group of MPS patients with a low likelihood of coronary artery disease. This approach may result in a post-test referral bias as a result of preferential selection of patients with a clear positive MPS examination for coronary angiography. Patients with slightly abnormal MPS findings that are not severe enough to justify motivate an angiogram will not be included. The results of this study are likely to better reflect the performance of the software packages in clinical routine.

### Reference classification

The use of coronary angiography as a gold standard has the advantage that it is an independent reference. Disadvantages include the patient-selection bias discussed above and the well-known lack of correspondence between a reduction in perfusion and the degree of coronary stenosis assessed from an angiogram in many cases. We therefore decided to use expert readings of the MPS images as our reference classifications, so as to avoid these disadvantages. In order to have the best possible reference classifications, we used three very experienced experts, each of whom had over 25 years' experience of nuclear cardiology. They separately classified all MPS images visually and did not use any quantification software in order not to be biased towards the results of that specific program.

We used three observers and applied the majority rule in order to minimize the effects of observer variability. All three experts agreed regarding infarction and ischemia in 83% and 71% of the cases, respectively. This illustrates that visual interpretation is subject to inter-observer variability, even for very experienced physicians. This is the motivation for the development of automatic software packages, to make the interpretations more standardized. In a study by Lindahl et al., three physicians separately classified 135 MPS studies twice without a computer-assisted diagnosis (CAD) system and thereafter twice using the advice of a CAD system [1]. They used a four-grade scale to classify the studies regarding presence of coronary artery disease in the LAD and LCX/RCA territories. Without the advice of the CAD system, they made the same classification for the same MPS study in 72% of the cases and with the CAD system in 82% of the cases. Those results showed that they improved their consistency with a CAD system and this was also the case for the most experienced of the three physicians, who had 20 years' experience of interpreting MPS studies.

Interpretation of coronary angiograms is also subject to observer variability. Banerjee et al. measured the agreement between independent assessments by two cardiologists in 209 coronary angiograms [13]. They found agreement regarding coronary disease to be 69% for the left circumflex artery, 82% for the right coronary and 89% for the left anterior descending arteries. This problem of variability needs to be addressed, regardless of whether coronary angiography or MPS is used as the reference. The gold standard was based on one experienced cardiologist for the angiographic evaluation in the study by Guner [7], and experienced physicians, who interpreted all coronary angiograms visually, in Wolak's study [6].

### Normal databases

We used the normal databases provided in the software packages. Both Wolak et al. [6] and Guner et al. [7] found that the application of an institutional normal database did not significantly improve the performance of the software. Furthermore, to our knowledge, most clinical users of the software packages use a normal database provided by the vendor and not their own institutional normal database. We wanted the results to reflect the performance of the software packages in clinical routine and we therefore used the same normal databases that are available to other users of the software packages.

The custom normal database used by Wolak et al. consisted of 50% patients with a body mass index (BMI) of over 30 and in their angiographic group 45% had a BMI greater than 30 [6]. In this study, 18% of the patients had a BMI greater than 30. Thus, adopting an American normal database for analyzing a patient population with low prevalence of subjects with high BMI may be critical, but that is again to our knowledge the most common way to use these software packages. In order to mimic the clinical routine of a European MPS clinic, we evaluated the three software packages with their American normal databases and a gold standard based on a European team of physicians. We therefore feel that this type of study is of interest to clinicians at European MPS clinics.

### Limitations

This study is focused on the diagnostic performance of commonly used criteria for abnormality for the three software packages 4DM, ECTb, and QPS. In clinical practice, the SSS or TDE value and the location of the blackout or the segments with abnormal scores is also important for the physician interpreting the study. The abnormal case in Figure 1A illustrates that the degree of disease can differ substantially (SSS range from 4 to 21 and TDE from 7% to 47%) also in cases that are classified as true positive for all software packages. The normal case in Figure 1C illustrates that the location of blackouts can differ. QPS indicates a septal defect, 4DM indicates a lateral defect, and ECTb both a septal defect and a lateral defect. The three software packages have different approaches to handle the difficult basal region, with different methods for delineation of the left ventricle (Figure 1). Also, differences in the normal databases and definitions of abnormal pixel values probably explain the different quantification results.

Thus there are clinically very important differences between the software packages regarding the size and location of the abnormalities that are not assessed in this study or in the studies of Wolak et al. [6] or Guner et al. [7].

This study was conducted without attenuation correction. This is not routinely performed in our department, but it is an essential tool in quantitative analysis and is likely to become more widely used. Possible attenuation artifacts mimicking a perfusion abnormality were present both for the three experienced readers involved in the reference classification and in the analysis of the software packages. The accuracies of the software packages presented in this study would therefore probably not be significantly influenced by attenuation artifacts. This is more likely to happen if an independent gold-standard method such as coronary angiography is used. Thus, attenuation artifacts probably only had a minor influence on the results of this study, since both the experts produced the reference classifications and the software packages, used non-corrected normal databases, and analyzed non-corrected images.

## Conclusion

There are considerable differences in performance between the three software packages with 4DM showing the best performance and ECTb the worst. These differences in performance should be taken in consideration when software packages are used in clinical routine or in clinical studies.

## Competing interests

Lars Edenbrandt is shareholder in EXINI Diagnostics AB, Lund, Sweden, a company that provides decision support systems for myocardial perfusion imaging.

## Authors' contributions

LJ participated in the design of the study, performed the analysis of the toolboxes and drafted the manuscript. ML, JM, and SES performed the reference classifications. MO performed the statistical analysis. LE conceived of the study, and participated in its design and coordination and helped to draft the manuscript. All authors read and approved the final manuscript.
